# Tuning the Threshold Voltage in Organic Thin-Film Transistors by Local Channel Doping Using Photoreactive Interfacial Layers

**DOI:** 10.1002/adma.201002912

**Published:** 2010-10-07

**Authors:** Marco Marchl, Matthias Edler, Anja Haase, Alexander Fian, Gregor Trimmel, Thomas Griesser, Barbara Stadlober, Egbert Zojer

**Affiliations:** Institute of Chemistry of Polymeric Materials, University of LeobenOtto Glöckel-Strasse 2, 8700 Leoben (Austria); Institute for Nanostructured Materials and Photonics, Joanneum ResearchFranz Pichler Straße 30, 8160 Weiz (Austria); Institute for Chemistry and Technology of Materials, Graz University of TechnologyStremayrgasse 9, 8010 Graz (Austria); * Institute of Solid State Physics, Graz University of TechnologyPetersgasse 16, 8010 Graz (Austria)

**Keywords:** organic field effect transistors, organic electronics, doping, polymeric materials

In recent years, organic thin-film transistors (OTFTs) have attracted a great deal of attention due to their potential applications in low cost sensors,^1^ memory cards,^2^ and integrated circuits.^3^ Great efforts are under way to design OTFTs with high performance, high stability, high reproducibility, and low cost.^4^ Two of the most crucial device parameters are the charge carrier mobility and the threshold voltage (*V*_Th_). Concerning the mobility, the main goals for most applications is its maximization.^5^ For *V*_Th_, the situation is more complex: for example, for integrated circuits it would be desirable to tune *V*_Th_ over a broad range,^6^ e.g., for inverter applications. In silicon technology, complementary circuits that consist of p-channel and n-channel transistors are typically used.^7^ There have been many attempts to adapt this technology to OTFTs and fabricate organic complementary inverters.^2^,^8^,^9^ They, however, suffer from poor n-type transistor performance and/or air instability of n-type semiconductor materials. An alternative approach is the adaptation of unipolar depletion-load inverters enabling simplified processing, even if they do not provide the low power consumption and the simple circuit design intrinsic to complementary logic.^10^,^11^ Depletion-load inverters consist of an enhancement-mode driver transistor and a depletion-mode load transistor and can be realized using only p-type OTFTs. So far there have been attempts to achieve this target by using a level shifter^12^,^13^ or a dual gate structure.^14^ The main objective is to find a reproducible method to realize driver and load transistors with equivalent device characteristics (in particular mobilities), but different *V*_Th_ values.

Over the years, a number of methods have been developed to tune threshold voltages. For example, oxygen plasma treatment of an organic gate dielectric (parylene) results in a large shift of *V*_Th_ to more positive values.^15^ This shift is related to the generation of charged surface states at the dielectric-semiconductor interface but the exact mechanism is only poorly understood. The same holds for UV-ozone treatments^16^ of the parylene and for the UV treatment of a poly-4-vinylphenol layer,^17^ where the latter has been used to realize and eventually to optimize depletion-load inverters. *V*_Th_ can also be tuned by changing the capacitance of the dielectric,^18^ by inserting a polarizable layer into the dielectric,^19^,^20^,^21^ or by using this polarizable layer as an encapsulation.^22^ With the latter methods, *V*_Th_ can be controllably and reversibly shifted over a wide range. The drawback of such approaches is, however, that a high “programming” voltage is needed to tune *V*_Th_. Another possibility to tune *V*_Th_ is by insertion of self-assembled monolayers^23^,^24^ or chemically reactive thin layers.^25^,^26^ The latter have been shown to tune *V*_Th_ by a local channel doping using acid groups that can be combined with de-doping reactions using bases. The disadvantage of the latter methods is that a local patterning of threshold voltages is not straightforward. Such a patterning is, however, a prerequisite for the realization of integrated electronic circuits.

Here, we significantly refine the concept of chemical channel doping by replacing the covalently bonded silane layers bearing sulfonic acid groups used in Refs. 25,26 with photoacid generator polymers.^27^ The goal hereby is to use them as an interface-modification layer (see Figure 1a), whose properties can be patterned photochemically, because, in contrast to the molecules used previously^25^,^26^, the acid group is formed only upon illumination. This paves the way for a photolithographic patterning of the interfacial doping and, thus, for controlling which transistors in a circuit operate in depletion or in enhancement mode. In other words, it enables an accurate local control of *V*_Th_ through the illumination dose by a method that is fully compatible with lithographic techniques omnipresent in conventional semiconductor industry.

**Figure 1:**
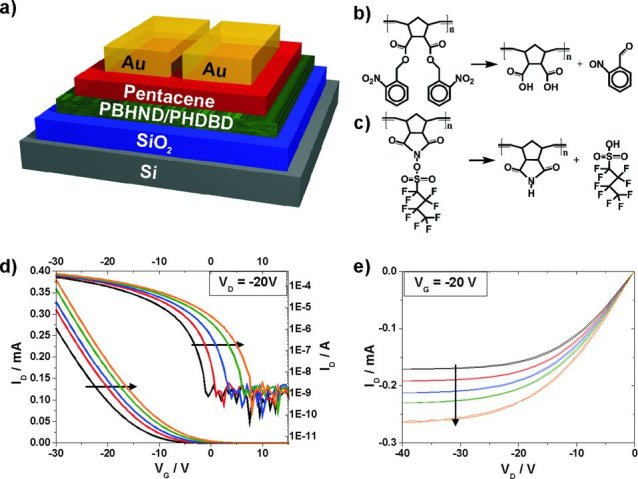
a) Schematic OTFT setup with the photoreactive layer embedded between dielectric and active layer. b,c) Dominant photoreactions of PBHND and PHDBD under UV illumination. d) Transfer and e) output characteristics for a representative series of PBHND-OTFTs with illumination times of 0, 2, 3, 4, and 5 s; the arrows denote increasing illumination time. For the sake of clarity, only the off to on (forward) sweeps of the transfer characteristics are shown. The hysteresis is small and increases slightly with illumination time to Δ*V*_G_ = 2 V at *I*_D_ = 0.10 mA for the longest illumination time included in the plot. The full transfer characteristics including forward and backward gate voltage sweep and the characteristics for longer illumination times are included in the Supporting Information. As far as the output characteristics are concerned, forward and backward sweeps coincide.

To show the versatility of our approach, we have synthesized two qualitatively different polymers, namely, poly(endo,exo-bicyclo[2.2.1]hept-5-ene-2,3-(2-nitrobenzyl) dicarboxylate) (PBHND) and poly-[(endo,exo-N-hydroxy bicyclo[2.2.1]hept-5-ene-2,3-dicarboximide perfluoro-1-butanesulfonate)-co-(endo,exo- bicyclo[2.2.1]hept-5-ene-2,3-dicarboxylic acid, dimethyl-ester)] (PHDBD). The chemical structures and the main reaction products upon UV illumination are shown in Figure 1b,c. From PBHND, a carboxylic acid is formed that remains chemically linked to the polymer backbone, while in PHDBD a sulfonic acid is part of the leaving group. Both photochemical reactions can be followed by IR spectroscopy (see Supporting Information).

In the device, the deprotonation of the acidic groups due to the reaction with the organic semiconductor results in the formation of a space-charge region at the interface. This gives rise to a threshold voltage shift, since mobile holes have to compensate the conjugated bases. This has been shown by drift-diffusion based modelling.^28^,^29^ From the data in 29 it can be estimated that the deprotonation of only a few percent of the acid groups at the interface is sufficient to shift *V*_Th_ by several tens of volts. An alternative way to understand the underlying process suggested for a polythiophene active layer^25^ is to view it as acid doping, in analogy with the situation in PEDOT/PSS.^30^ Indeed, a photoinduced protonation of pentacene by acids has already been shown, albeit only at temperatures around 3.5 K with the product being thermally unstable.^31^ Consequently, the occurrence of stable protonated pentacene molecules appears unlikely for the present devices and the actual fate of the protons needs to be investigated further.

The results in Figure 1d show that varying the illumination time allows tuning the threshold voltage over several volts by increasing the UV illumination time from 0 to 5 seconds in a PBHND-containing OTFT. The shape of the curves is essentially the same for the different illumination times; in particular the slopes (and thus the mobilities) of the OTFTs remain constant. Also the drain current in the output characteristics (Figure 1e) increases with illumination, consistent with an increased channel doping. The hysteresis of the transistors becomes somewhat larger but remains small even after 5 s of illumination. The gate voltage at a drain current of *I*_D_ = 0.10 mA changes by Δ*V*_G_ = 2 V. Also the off-current remains in the range of nA and is barely altered by the UV treatment. The devices, for which the data are shown in Figure 1e, were kept in an argon glove box throughout all process steps so no detrimental effects due to contact with O_2_ or H_2_O occured.

While the *V*_Th_ shifts shown in Figure 1d are sufficient for the realization of a depletion load inverter (vide infra), the impact of longer illumination times (see left part of Figure 2) was also explored. The time scales for illumination cannot be directly compared, as different lamps with different emission spectra were used for the two different polymers (see Experimental Section). The observed threshold voltages changes reach close to 50 V both for PBHND and PHDBD interfacial layers. However, the threshold values without illumination differ significantly between the two photoacid generator polymers (only the PHBDB devices are actually switched from enhancement to depletion mode operation). Moreover, the *V*_Th_ shifts for PHDBD interfacial layers are not stable for repeated measurement cycles, as shown in the right plot in Figure 2.^32^

**Figure 2:**
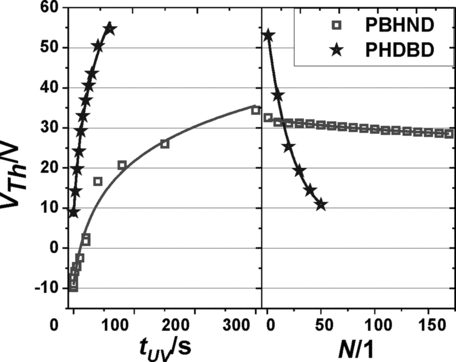
Left: Evolution of the threshold voltage as a function of illumination time for OTFTs containing PHBND (open squares) and PHDBD (filled stars) interfacial layers. The logarithmic fit is a guide to the eye. Right: Stability of the threshold voltage shift in PHDBD- and PHBND-containing OTFTs as a function of the number of measurement cycles. The measurements were performed at varying time intervals. The fact that a smooth curve was obtained indicates that only the measurement cycle itself adversely affects the device characteristics, i.e., the decrease in *V*_Th_ is essentially a bias stress effect. For the sake of clarity a symbol is plotted for only every tenth measurement point. The exponential fits are guides for the eye.

For the PBHND-based OTFTs, even after more than 200 measurements the threshold voltage did not shift back considerably. The small decrease during the first measurements was mostly due to a decrease of hysteresis in these devices. The hysteresis is at least in part a result of the need to briefly transport the devices through air before the illumination (which, again, happened under inert atmosphere). One also has to keep in mind that the chosen measurement range for the device characteristics (from −60 V to 60 V) induces a significantly larger amount of bias stress than in any inverter-based application. The small offset between the final data point in the left plot and the first data point in the right plot is a consequence of using devices from different samples. The difference in device stability for the illuminated PBHND- and PHDBD-containing devices can be attributed to the different positions of the acids formed in the photochemical process. As mentioned above, in PBHND the acid is linked directly to the polymer backbone, while it is on the leaving group in PHDBD.

An observed disadvantage of extended illuminations is that, in addition to the *V*_Th_ shift, it results in some deterioration of the device characteristics. These effects, however, play a role only for illumination times far beyond the 3 s needed for the optimum inverter operation. They include an increase of the off current, a larger hysteresis, and a deterioration of the drain current at large negative gate bias. A detailed discussion can be found in the Supporting Information. All measurements (threshold voltage shift and stability) for the PHDBD devices were reproduced for several samples. The PHBND measurements were reproduced for two batches of freshly synthesized polymer and for various samples within one batch.

To verify that the observed effects are indeed a consequence of interfacial acid doping, two test experiments were performed. First, the devices were exposed to a strong base (NH_3_ as in Refs. 25,26). As expected, this treatment results in a neutralization of the acid, i.e., a de-doping of the channel, and after 10 min of exposure to ammonia the devices were switched back to a situation similar to that prior to UV exposure. As a second test, devices containing no interfacial layer were also illuminated; here, even after 20 min of UV exposure no changes in the transfer and output characteristics were observed. More details about these experiments are included in the Supporting Information.

This provides us with all the tools necessary to perform the next step in the direction of integrated p-type organic electronic devices, which is the fabrication of a (tuneable) depletion-load inverter. The wiring diagram of such an inverter is plotted as an inset in Figure 3. The switch transistor is realized by a non-illuminated PBHND-containing device that has a negative threshold voltage of around *V*_Th_ = –10 V. The load transistor (also a device containing a PBHND interfacial layer) was illuminated in steps of 1 s in an argon glove box after the inverter wiring was realized. As seen in Figure 3, the inverter characteristic is very poor prior to illumination, the gain of the inverter is negligible, and the achievable output voltage at the low level is only −4 V. This is because the non-illuminated load transistor is normally off and works in enhancement mode. By increasing the illumination time of the load transistor and shifting its threshold towards the positive voltage regime, the inverter characteristic improves significantly. The turn-on voltage of the inverter is shifted to more negative values and the gain of the inverter increases. After illuminating the load transistor for 3 s, an optimum value of *V*_Th,load_ with respect to the threshold voltage of the switch-transistor is reached, resulting in a steep inverter transition with a maximum gain of about 40 (see bottom graph in Figure 3). Further increasing the illumination time results in a deterioration of the inverter performance. At this point it should be noted that no attempts for optimizing the inverter characteristics other than tuning *V*_Th,load_ were made. Therefore, further significant improvements can be expected by adapting the width-to-length ratio of the channel between the load and switch and by optimizing the performance of individual transistors with respect to mobility, gate leakage, etc.

**Figure 3:**
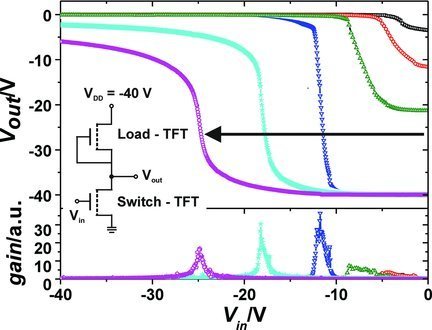
Top: Inverter characteristics with differently illuminated load TFTs (illuminated for 0, 1, 2, 3, 4, and 5 s); the arrow shows the trend for increasing illumination times. Bottom: the corresponding gains of the inverters; inset: wiring diagram of a depletion-load inverter.

In conclusion, we have demonstrated an easy and reproducible way to switch OTFTs from enhancement to depletion mode by a photochemical reaction using photoacid generators as interfacial layers and demonstrated that this allows the fabrication of good quality depletion-load inverters with tunable characteristics. The suggested fabrication method can easily be adapted to monolithically integrated circuits by illuminating individual transistors through shadow- or photomasks when patterned gate electrodes are used (in this way simultaneously reducing leakage and parasitic effects).

## Experimental Section

The two polymeric photoacid generators poly(endo,exo- bicyclo[2.2.1]hept-5-ene-2,3-(2-nitrobenzyl)dicarboxylate) (PBHND) and poly-[(endo,exo- N-hydroxy bicyclo[2.2.1]hept-5-ene-2,3-dicarboximide perfluoro-1-butanesulfonate)-co-(endo,exo- bicyclo[2.2.1]hept-5-ene-2,3-dicarboxylic acid, dimethyl-ester)] (PHDBD) were synthesized following the previously described procedure,^33^ using nitrobenzylalcohol for monomer synthesis and a Grubbs initator for polymerization. Further details are given in the Supporting Information.

Highly p-doped silicon wafers with a 155-nm-thick layer of thermally grown SiO_2_ were purchased from Siegert Consulting e.K. (Aachen, Germany) All wafers were O_2_-plasma etched for 30 s and then rinsed in deionized H_2_O in an ultrasonic bath for 120 s. Subsequently, the photoreactive polymers PBHND and PHDBD were spin-cast from a solution in tetrahydrofuran (4mg mL^−1^) at 1000 rotations per minute (rpm) for the first 9 s and at 2000 rpm for the following 40 s. This yielded films of 35 nm thickness for PBHND and 10 nm thickness for PHDBD, as determined by X-ray reflectivity measurements. Afterwards, pentacene layers with an average thickness of 35 nm (measured with a quartz-microbalance) were evaporated at a base pressure of 1 × 10^−5^ mbar with the substrates held at room temperature. The first 5 nm were evaporated at a rate of 0.02 A s^−1^ and the subsequent 30 nm at a rate of 0.1A s^−1^. 50-nm-thick Au source and drain electrodes were deposited through a shadow mask at a base pressure of 4 × 10^−6^ mbar. The resulting channel length and width were 50 μm and 7 mm, respectively. On the left side of Figure 1 the whole device setup of the OTFTs is displayed.

The as-fabricated and fully functional OTFTs were exposed to UV light in helium atmosphere to avoid photo-oxidation of the polymers. In this context it needs to be mentioned that in another series of experiments it was found that when illuminating PBHND prior to pentacene deposition and subsequently exposing the devices to air before growing the semiconductor, the observed *V*_Th_ shifts were comparably small but the growth of the pentacene could be reproducibly tuned by the illumination time.^34^ This can, for example, be used for tuning the film mobilities and is possibly a consequence of the interaction of the acid groups with ambient humidity. Such processes were avoided in the present experiments.

Here, for the post-growth illumination, the PBHND TFTs were illuminated in a range from 1 s to 300 s with an intensity of *I*_UV_ = 3 W cm^−2^ with an EFOS (now EXFO, Mississauga, Canada) Novacure lamp. PHDBD TFTs were illuminated for 1 to 60 s with a 100 W polychromatic medium pressure mercury lamp from Heraeus (Hanau, Germany) in a Newport (Irvine, USA) model 66990 housing. For the latter experiments, the light intensity (power density) at the sample surface was measured with a spectroradiometer (Solatell (Stroud, UK), Sola Scope 2000TM, measuring range from 230 to 470 nm). The integrated power density for the spectral range 230nm-400 nm was 20.9 mW cm^−2^.

The devices were characterized immediately after the illumination in an argon glove box with a Parametric Analyzer (Agilent Technologies (Santa Clara, USA) – E5262A).^35^ Threshold voltages are extracted from the low-voltage (i.e., saturation) regime of the transfer characteristics as this is the most relevant voltage region for the inverter characteristics.^36^ This is useful here in spite of the issues arising from carrier-density-dependent mobilities. For the fabrication of the inverters, the devices were illuminated directly in the argon glove box. The wiring was performed externally through two Parametric Analyzers (HP4145, mb technologies (Grosswilfersdorf, Austria)). This was necessary, as substrates with common gate structures were used, due to which the switch and load transistor had to be fabricated on different substrates. There is, however, no fundamental reason, why the two transistors cannot be fabricated next to each other on the same substrate, with a phototuning of *V*_Th_ of the load transistor through a mask, when patterned gate structures are available.
