# Supplementary material for: Tuning the Threshold Voltage in Organic Thin-Film Transistors by Local Channel Doping Using Photoreactive Interfacial Layers
Source: Adv Mater. 2010 Oct 7;22(47):5361–5. doi: 10.1002/adma.201002912 (PMC3021719; doi:10.1002/adma.201002912)
Supplement: Supplementary file 1 [file adma0022-5361-SD1.pdf]

Copyright WILEY-VCH Verlag GmbH & Co. KGaA, 69469 Weinheim, Germany, 2010.

ADVANCED MATERIALS

Supporting Information

for *Adv. Mater.*, DOI: 10.1002/adma.201002912

Tuning the Threshold Voltage in Organic Thin-Film Transistors by Local Channel Doping Using Photoreactive Interfacial Layers

By Marco Marchl, Matthias Edler, Anja Haase, Alexander Fian, Gregor Trimmel,
Thomas Griesser,* Barbara Stadlober,* and Egbert Zojer*

Supporting Information

1) Synthesis of polymers
2) IR-data of the photoreactions
3) Dedoping with ammonia
4) Illumination effect on standard pentacene devices
5) Detrimental effects of longer illumination times

- **Synthesis of the polymers**

Endo,exo- bicyclo[2.2.1]hept-5-ene-2,3-(2-nitrobenzyl)dicarboxylate

Under inert atmosphere (2-nitrophenyl)methanol (2.42g,15.8 mmol) and pyridine (1.24 ml, 15.8 mmol) were dissolved in 40 mL dry CH_2Cl_2. Under ice cooling *endo,exo*[2.2.1]bicyclo-2-ene-5,6-dicarboxyclic acid chloride (1.56 g, 7.18 mmol) was slowly dropped into the reaction mixture, the cooling bath was removed, and the reaction mixture was stirred overnight at room temperature. The reaction mixture was filtered to remove the pyridine salt and extracted with dichloromethane. The organic layer was extracted with 3 × 20 mL 5% hydrochloric acid solution and 20 mL saturated sodium bicarbonate and dried with sodium sulphate. Column chromatography with cyclohexane/ethylacetate (10:1) was used to purify the product. The precipitate was dried in vacuo. Yield: 2.69g (83 %) of a white solid.

^{1}H-NMR: (δ, 400 MHz, 20°C, $CDCl_3$): 8.09 (m, 2H, ph^3); 7.60 (m, 2H, ph^4); 7.58 (m, 2H, ph^5); 7.49 (d, 2H, ph^6); 6.30; 6.11 (m, 2H, $nb^{5,6}$); 5.55-5.47 (m, 4H, O-CH_2-ph); 3.51 (m, 1H, nb^3); 3.36 (s, 1H, nb^4); 3.21 (s, 1H, nb^1); 2.83 (m,1H, nb^2); 1.67; 1.54 (d, 2H, nb^7) ppm.

^{13}C-NMR: (δ, 100 MHz, 20°C, $CDCl_3$): 173.6, 172.4 (COO); 137.71 (ph^2-NO_2); 137.62 135.16 ($nb^{5,6}$);133.74, 133.63 (ph^5); 131.98, 131.67 (ph^1); 129.26, 129.15 (ph^4); 128.87 (ph^6); 125.87; 125.02 (ph^3); 63.31, 63.54 (O-CH_2-ph); 48.67 (nb^2); 47.60 (nb^3); 47.57 (nb^1); 47.20 (nb^4); 45.78 (nb^7) ppm.
FTIR- (CaF_2, cm^{-1}): 1733; 1613; 1578; 1525; 1447; 1342; 1306; 1163.

Polymerisation

Poly(endo,exo- bicyclo[2.2.1]hept-5-ene-2,3-(2-nitrobenzyl)dicarboxylate)

To a solution of 450 mg (0.99 mmol) *endo,exo- bicyclo[2.2.1]hept-5-ene-2,3-(2-nitrobenzyl)dicarboxylate* in 4 mL dichloromethane 4.10 mg (4.95 μmol) $RuCl_2(PC_{y3})_2(CHPh)$

dissolved in 2 mL of dichloromethane were added. The reaction mixture was stirred at room temperature for 24h and then the reaction was stopped by adding 5 drops of ethyl-vinylether and the polymer was precipitated by dropping the solution into cold methanol. The precipitate was dried in vacuo.

Yield: 361 mg (80.2 %) of a white solid. GPC ($CHCl_3$): M_n = 44690 g/mol; PDI = 1.2; T_g = 57°C

^{1}H: NMR (400 MHz, 20°C, $CDCl_3$): 8.10-7.84 (s, 2H, ph^3); 7.67-7.26 (m, 6H, $ph^{4,5,6}$); 5,65-5.03 (m, 6H, CH=CH, -O-CH_2-); 3.50-2.64 (m, 4H, $nb^{1,2,3,5}$); 2.22-1.45 (m, 2H, nb^4) ppm. FT-IR (CaF_2, cm^{-1}): 1738; 1613; 1577; 1524; 1450; 1342; 1306; 1162; 1001; 971.

Poly-[(endo,exo- N-hydroxy *bicyclo[2.2.1]hept-5-ene-2,3-dicarbox*imide perfluoro-1-butanesulfonate*)-co-(endo,exo- bicyclo[2.2.1]hept-5-ene-2,3-dicarboxylic acid, di-methyl-ester)]*

To a solution of 100 mg (0.22 mmol) *endo,exo-* N-hydroxy *bicyclo[2.2.1]hept-5-ene-2,3-dicarbox*imide perfluoro-1-butanesulfonate and 45.75 mg (0.22 mmol) *endo,exo- bicyclo[2.2.1]hept-5-ene-2,3-dicarboxylic acid, di-methyl-ester)* in 4 mL dichloromethane 1.85 mg (2.18 µmol) (H_2IMes)(PCy_3)$(Cl)_2$Ru=CHPh dissolved in 2 mL of dichloromethane were added. The reaction mixture was stirred at room temperature for 24h and then the reaction was stopped by adding 5 drops of ethyl-vinylether and the polymer was precipitated by dropping the solution into cold methanol. The precipitate was dried in vacuo.

Yield: 120 mg (82.3 %) of a white solid.

GPC ($CHCl_3$): M_n = 67110 g/mol; PDI = 2.9; T_g = 136 °C.

^{1}H: NMR (400 MHz, 20°C, $CDCl_3$): 5.75-5.08 (m, CH=CH, 4H), 3.80-3.54 (m, CH_3, 6H); 3.46-2.55 (m, 8H, $nb^{1,2,3,5}$); 2.20-1.36 (m, 4H, nb^4) ppm.

FT-IR (CaF_2, cm^{-1}): 2958; 1756; 1447; 1350; 1234; 1202; 1144.

- **IR-data of the polymers**

1) PBHND

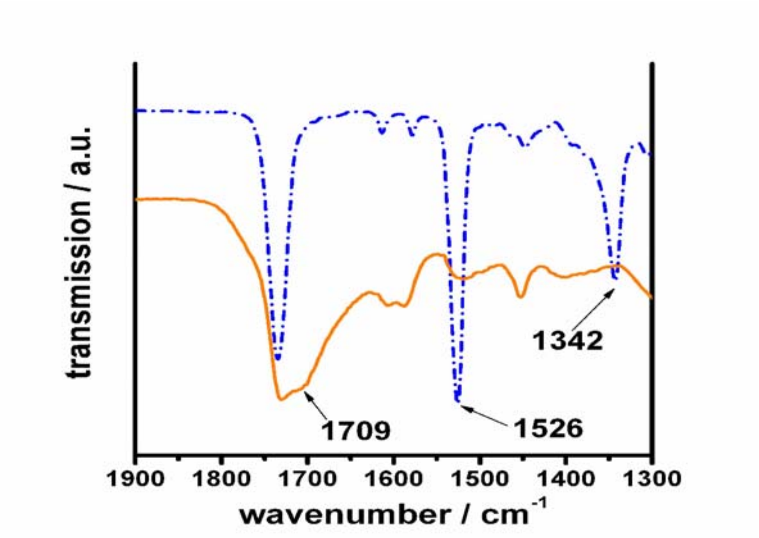

Fig. S1: IR-data of illuminated (orange)/non illuminated (blue) films of PBHND

The changes in the IR spectra of a PBHND film upon irradiation are depicted in Fig. S1. They confirm the occurrence of the photoreaction: The bands associated with the nitro groups at 1526cm^{-1} and 1342cm^{-1} vanish and a new band at 1709cm^{-1} appears that is related to the carboxylic acid.[1] To exclude that a heating of the film (e.g., during pentacene evaporation) has any consequence on its composition, also PBHND films heated to 100°C for 1h were investigated, but no changes in the IR-spectra were observed.

2) PHDBD

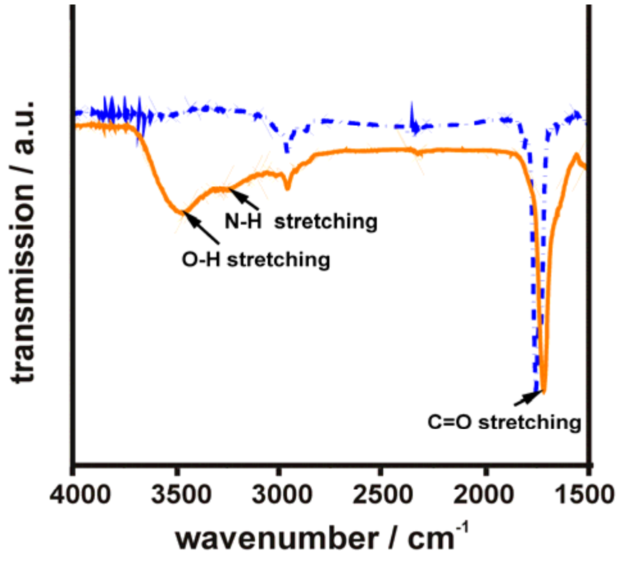

Fig. S2: IR-data of (orange)/non illuminated (blue) films of PHDBD.

Also the changes in the IR spectra of the PHDBD-film upon irradiation, which are depicted in Fig. S2, confirm the occurrence of the photoreaction: The band associated with the sulfonic acid groups at 3480 cm^{-1} vanishes and a new band at 3250 cm^{-1} appears that corresponds to the NH-stretching of the imid-group.[1] The CO stretching shifts from 1750 cm^{-1} to 1710 cm^{-1}

upon illumination. Also here, to exclude that a heating of the film (e.g., during pentacene evaporation) has any consequence on its composition, PHDBD films heated to 100°C for 1h were investigated, but again no changes in the IR-spectra were observed.

- **The effect of exposure to ammonia on the transfer characteristics**

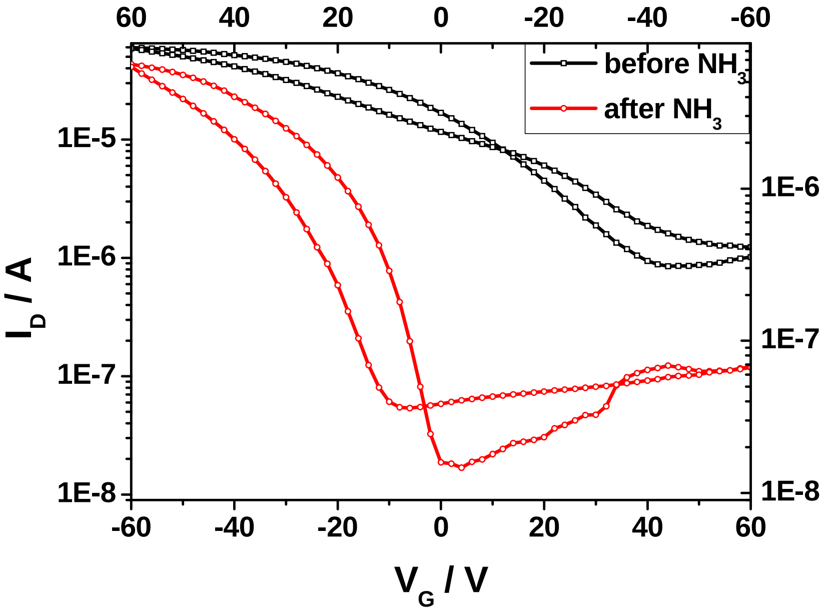

Fig. S3: Transfer curves for a PBHND *OTFT before and after the reaction with ammonia.*

A PBHND transistor that was illuminated for 60 sec and then the transfer curve was recorded (black line in Fig. S3). Afterwards, the transistor was exposed to pure ammonia gas for ten minutes and then measured again in inert atmosphere (red line in Fig. S3). As can bee seen the doping effect completely vanishes and the transistor is switched back to accumulation mode. The threshold voltage after the reaction with ammonia is even more negative than for not illuminated devices. A part of the high hysteresis in the transfer curve as well as the high off-currents after the reaction with ammonia result from the fact that during the process, the transistors had to be transported through air earlier.

- **The effect of UV-illumination for devices not containing a reactive layer**

To further ensure that the photoreaction of the polymer is the reason for the threshold-voltage shift in the OTFTs, also plain transistors, i.e., OTFTs without any photoreactive layer, were illuminated with UV-light under the same experimental conditions as all the other TFTs. As can be seen in Fig. S4, the transfer curves for a non-illuminated and an illuminated device (60 sec UV-illumination time) show hardly any difference. This confirms that the photoreaction of the polymers is the crucial process inducing the threshold-voltage shift.

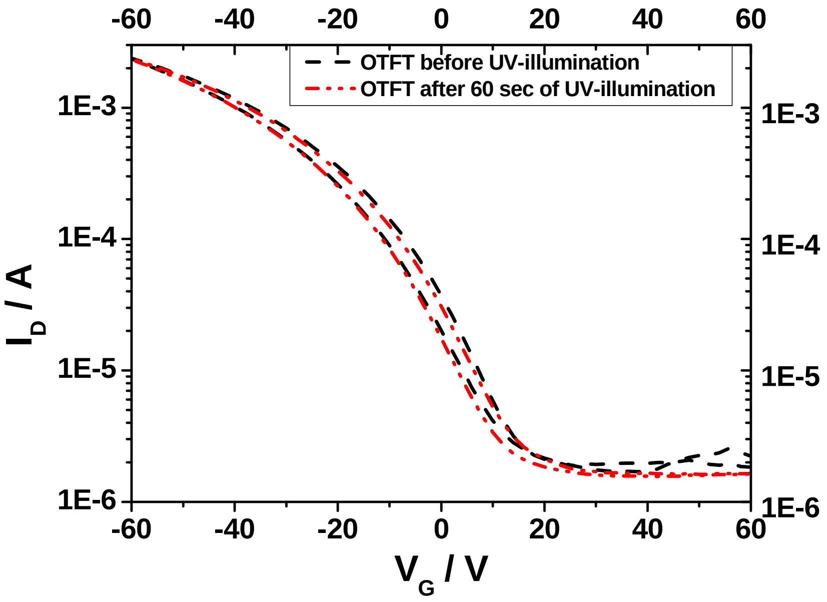

Fig. S4: Transfer curves for a plain OTFT before and after illumination with UV-light. The higher off-currents compared to Fig. S5 are attributed to an exposure of the current devices to air during the fabrication process, which has been strictly avoided for the devices in Fig. S5 and for the inverters in Figure 3 of the main manuscript.

- **A closer look at the transfer curves for long illumination times**

Fig. S5 (PBHND) shows the same transfer curves as in Fig 1 of the main paper but this time also the on-to-off sweeps are included to show the hysteresis. Fig. S6 (PBHND) compares a non-illuminated device with a device that was illuminated for 300 seconds. Fig. S7 shows an illumination series for the PHDBD devices (ranging from 0 to 60 seconds). When looking at the transfer curves in Fig S5, Fig. S6, and Fig. S7, the following effects can be identified.

1) Increase of the off-current for long illumination times (Fig. S6 & Fig. S7):
The increase of the off-current is accompanied by an increase of the gate leakage current. Apparently, the UV-illumination damages the $Si0_2$ dielectric.
2) Increase of the substhreshold swing (Fig. S6 & Fig. S7):
Following the equation of Unni et al.[S4] the upper limit of the trap density N_{max} can be related to the subthreshold swing.

$$N_{\max} \approx [\frac{qS\log(e)}{kT} - 1]\frac{C_{Ox}}{q}$$

Here, q is the electronic charge and S the subthreshold swing in V decade^{-1}. k is Boltzmann's constant, C_{ox} the capacitance per area of the gate dielectric and T the temperature.
This implies that the increased sub-threshold swing for long illumination times is the consequence of a formation of traps through the illumination with UV-light.
3) Increase of hysteresis (Fig. S5 & Fig. S6 & Fig. S7):
The effect of hysteresis in OTFTs can also be associated with hole traps.[5] Thus, the increase of the hysteresis with increasing illumination time can also be explained by the photogeneration of traps in the semiconductor or at the surface of the gate dielectric. For the sake of good visibility the on-to-off sweep was omitted in Fig. S6, but the hysteresis and the off-current increase dramatically with increasing UV-illumination times. (ΔV_G = 25V at I_D = 0.04 mA after 60 seconds of illumination)

4) Decrease of the on current for long illumination times in the case of PHDBD devices (Fig. S7):

Similar characteristics have been described earlier,[S2,S3] and were explained by a continuous increase of the contact resistance. Possibly, the illumination with UV light (in this special spectral range) has an adverse effect on the contact between gold and pentacene.

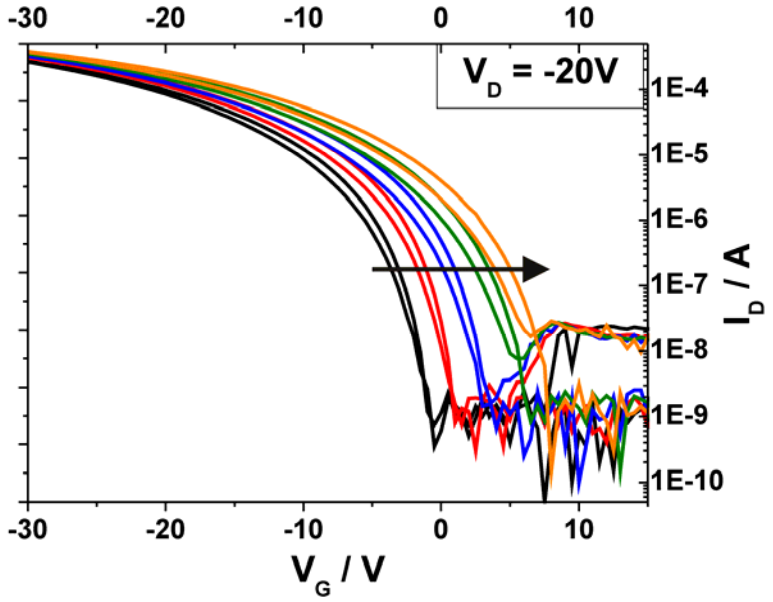

Fig. S5: Transfer curves for a representative illumination series of PBHND - TFTs for short illumination times (corresponding to Fig.1 in the main paper). The illumination times were 0,2,3,4 and 5 seconds. The arrow shows the direction of increasing illumination time.

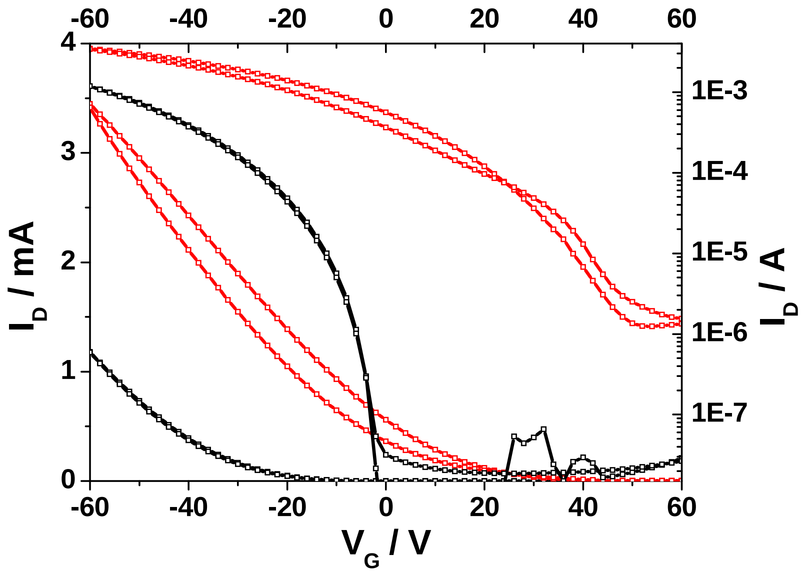

Fig. S6: Transfer curves of a non- illuminated PBHND TFT (black curve) and a PBHND TFT that was illuminated for 300 seconds (red curve) measured at V_D = -45 V. The slightly higher off-currents compared to Fig. S5 are attributed to an exposure of the current devices to air during the fabrication process, which has been strictly avoided for the devices in Fig. S5 and for the inverters in Figure 3 of the main manuscript.

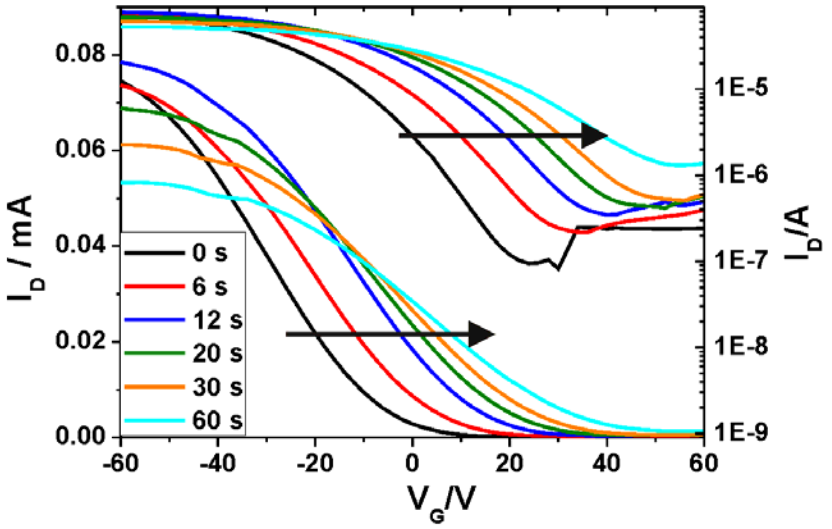

Fig. S7: Tranfer curves for a representative illumination series of PHDBD-TFTs (ranging from 0 to 60 seconds) measured with V_D = -45 V. The arrows indicate the direction of increasing illumination times. For the sake of good visibility the on-to-off sweep was omitted in this figure. The higher off-currents compared to Fig. S5 are attributed to the different interfacial layer and to an exposure of the current devices to air during the fabrication process, which has been strictly avoided for the devices in Fig. S5 and for the inverters in Figure 3 of the main manuscript.

.

[S1] G.Socrates, Infrared and Raman Characteristic Group Frequencies: Tables and Charts, 3rd ed. Wieley: New York, 2001

[S2] M. Mottaghi, G. Horowitz ,*Org. Elect*. **2006**, *7*, 258.

[S3] S. Mansouri, M. Mahdouani, A. Oudir, S. Zorai, S. Ben Dkhil, G. Horowitz, R. Bourguiga, *Eur. Phys. J. Appl. Phys.* **2009**, *48*, 30401.

[S4] K.N. N. Unni, S. Dabos-Seignon, J.-M. Nunzi, *J. Mater. Sci*. **2006**, *41*, 317

[S5] C. Ucurum, H. Goebel, F. A. Yildirim, W. Bauhofer, W. Krautschneider, *Journ. Appl. Phys*. **2008**, *104*, 084501
